# Examination of Sarcopenia with Obesity as a Prognostic Factor in Patients with Colorectal Cancer Using the Psoas Muscle Mass Index

**DOI:** 10.3390/cancers16193429

**Published:** 2024-10-09

**Authors:** Kengo Haruna, Soichiro Minami, Norikatsu Miyoshi, Shiki Fujino, Rie Mizumoto, Yuki Toyoda, Rie Hayashi, Shinya Kato, Mitsunobu Takeda, Yuki Sekido, Tsuyoshi Hata, Atsushi Hamabe, Takayuki Ogino, Hidekazu Takahashi, Mamoru Uemura, Hirofumi Yamamoto, Yuichiro Doki, Hidetoshi Eguchi

**Affiliations:** 1 Department of Innovative Oncology Research and Regenerative Medicine, Osaka International Cancer Institute, Osaka 545-0871, Japan; kharuna@gesurg.med.osaka-u.ac.jp (K.H.); sminami@gesurg.med.osaka-u.ac.jp (S.M.); sfujino@gesurg.med.osaka-u.ac.jp (S.F.); rmizumoto@gesurg.med.osaka-u.ac.jp (R.M.); rhayashi@gesurg.med.osaka-u.ac.jp (R.H.); skato@gesurg.med.osaka-u.ac.jp (S.K.); 2 Department of Gastroenterological Surgery, Graduate School of Medicine, Osaka University, Osaka 565-0871, Japan; ytoyoda@gesurg.med.osaka-u.ac.jp (Y.T.); mtakeda@gesurg.med.osaka-u.ac.jp (M.T.); ysekido@gesurg.med.osaka-u.ac.jp (Y.S.); tsuyoshihata@gesurg.med.osaka-u.ac.jp (T.H.); ahamabe@gesurg.med.osaka-u.ac.jp (A.H.); togino@gesurg.med.osaka-u.ac.jp (T.O.); muemura@gesurg.med.osaka-u.ac.jp (M.U.); hyamamoto@gesurg.med.osaka-u.ac.jp (H.Y.); ydoki@gesurg.med.osaka-u.ac.jp (Y.D.); heguchi@gesurg.med.osaka-u.ac.jp (H.E.); 3 Department of Surgery, Osaka Police Hospital, Osaka 543-0035, Japan; htakahashi@oph.gr.jp

**Keywords:** sarcopenia, obesity, colorectal cancer

## Abstract

**Simple Summary:**

This study assessed the prognostic impact of sarcopenic obesity (SO) in colorectal cancer patients. Among 211 sarcopenic patients, those with obesity (SO group) demonstrated significantly shorter cancer-related relapse-free survival (CRRFS) compared to their non-obese counterparts (non-SO group). While cancer-specific survival (CSS) was also poorer in the SO group, this difference did not reach statistical significance. Additionally, there was no significant difference in overall survival (OS) between the two groups. Multivariate analysis identified sarcopenic obesity, elevated CEA levels, and unfavorable histological types as independent predictors of poor CRRFS. These findings underscore the importance of routine assessment of both sarcopenia and obesity in clinical practice. Moreover, they highlight the potential benefits of interventions aimed at increasing muscle mass and reducing visceral fat to enhance patient outcomes.

**Abstract:**

**Background**: Sarcopenia, the age-related loss of muscle mass, is a negative prognostic factor in gastrointestinal cancer. Sarcopenia combined with visceral obesity (sarcopenic obesity) is associated with poor outcomes. We explored the influence of obesity and other factors on the prognosis of patients with colorectal cancer diagnosed with sarcopenia. **Methods**: We enrolled 211 patients with colorectal cancer diagnosed with preoperative sarcopenic obesity who underwent radical resection at Osaka University Hospital between January 2009 and January 2012. Muscle mass was assessed using the psoas muscle mass index. Obesity was evaluated by measuring the visceral fat area in the umbilical region. Patients were categorized into two groups: sarcopenia with obesity (SO) and sarcopenia without obesity (non-SO). Overall survival, cancer-specific survival, and cancer-related relapse-free survival (CRRFS) were compared between the two groups. Patient characteristics, including age, sex, body mass index, serum albumin, C-reactive protein, tumor markers, prognostic nutritional index (PNI), modified Glasgow prognostic score (mGPS), and geriatric nutritional risk index (GNRI), were also analyzed. **Results**: CRRFS was significantly shorter in the SO group than in the non-SO group (*p* = 0.028). PNI, mGPS, and GNRI were not identified as significant prognostic factors for CRRFS. Multivariate analysis highlighted sarcopenic obesity, elevated carcinoembryonic antigen levels, and unfavorable histological types as significant predictors of poor CRRFS outcomes. **Conclusions**: Sarcopenic obesity is an independent predictor of poor prognosis in patients with CRC. Thus, interventions aimed at increasing muscle mass and reducing visceral fat could potentially improve the prognosis of these patients.

## 1. Introduction

Sarcopenic obesity (SO) is a clinical condition characterized by the coexistence of obesity with low muscle mass and function. Sarcopenia is a progressive and generalized decrease in muscle mass due to aging, reduced activity and nutritional intake, and inflammation [1]. In patients with cancer, cancer-induced inflammation commonly leads to muscle catabolism and altered energy metabolism, making sarcopenia a common complication [2]. Further, sarcopenia is highly correlated with the risk of severe toxic effects from chemotherapy and associated body composition changes [3]. Some studies have reported a correlation between sarcopenia and poor prognosis in patients with colorectal cancer (CRC) [4,5,6]. Obesity, another feature of SO, is closely related to an increased risk of CRC [7] and is associated with increased all-cause mortality [8].

Maintaining adequate muscle mass requires sufficient protein intake [9]. However, in obese patients, protein intake may be insufficient, which can exacerbate the decline in muscle mass and function associated with sarcopenia [10]. This highlights the potential risk of inadequate protein consumption in obese populations, leading to poor muscle health and contributing to sarcopenic obesity.

As explained above, sarcopenia and obesity are both known to be associated with important metabolic derangements; however, the extent to which their combination produces synergistic effects and whether sarcopenic obesity may be considered a syndrome remain unknown.

The prevalence of obesity is increasing worldwide [11], and SO is becoming an increasingly relevant health concern. This increasing trend is related to the global rise in obesity, the growing number of patients with cancer and high body weight at diagnosis, and the increase in specific obesity-associated cancers. Given the role of protein in muscle maintenance and the potential for low protein intake in obese individuals, addressing nutritional deficits may be crucial in managing sarcopenic obesity.

Given this background, the aim of this study was to evaluate the impact of obesity and other factors on the prognosis of CRC patients with sarcopenia.

## 2. Methods

### 2.1. Patient Population

Our study included patients with CRC who underwent radical resection at our hospital between January 2009 and January 2012 in accordance with the inclusion and exclusion criteria.

The inclusion criteria were as follows: any age and sex, presence of medical files at our hospital, and the availability of abdominal CT scan data covering the entire abdomen and the third lumbar spine level conducted prior to treatment.

The exclusion criteria included distant metastases, patients who had undergone surgery for recurrence, and patients who had undergone transanal endoscopic microsurgery (TEM).

Patients were defined as sarcopenic if their psoas muscle mass index (PMI) was below the cutoff value and as obese if their visceral fat area (VFA) exceeded the cutoff value. After excluding patients without sarcopenia, the patients were divided into two groups: the sarcopenic obesity group, including patients with both sarcopenia and obesity, and the non-sarcopenic obesity group, which included patients with sarcopenia but not obesity.

The cutoff value for PMI was set at 6.36 cm^2^/m^2^ for men and 3.92 cm^2^/m^2^ for women. This was the cutoff value proposed in the Japanese Society of Hepatology criteria for determining sarcopenia in liver disease, defined as the mean value of PMI minus twice the standard deviation for healthy people under 50 years of age, as the standard for low skeletal muscle mass in Japanese people.

The cutoff value of the VFA was set at 100 cm^2^ for both men and women as adopted by the Japan Society for the Study of Obesity in its Obesity Treatment Guidelines, 2022.

The detection and diagnosis of sarcopenic obesity is a complicated procedure for which there are not sufficient tools and cutoff criteria, especially in oncology patients [12]. While various criteria for sarcopenia and obesity have been used in numerous international studies, most of these are based on Western populations, and it remains unclear whether these criteria are appropriate for Japanese individuals. Therefore, in our study, we adopted the criteria set by the Japan Society of Hepatology and the Japan Society for the Study of Obesity. Additionally, for obesity in this study, we used the definition of visceral fat obesity.

### 2.2. Imaging Analysis

The PMI was calculated by dividing the sum of the areas of the bilateral iliopsoas muscles at the third lumbar level (cm^2^) by the square of the height (m^2^). Obesity was evaluated using the VFA (cm^2^) at the umbilical level. The iliopsoas muscle and visceral fat areas were measured using the three-dimensional image analysis system Volume Analyzer SYNAPSE VINCENT (FUJIFILM Medical Co., Ltd., Tokyo, Japan) in digital imaging and communication in medicine (DICOM) format.

### 2.3. Examination Factors

The primary endpoints of overall survival (OS), cancer-specific survival (CSS), and cancer-related relapse-free survival (CRRFS) were compared between the sarcopenia-obese and non-sarcopenia-obese groups. We analyzed the patient characteristics, including age, sex, body mass index (BMI), serum albumin, C-reactive protein (CRP), preoperative tumor markers, tumor localization, histopathology, tumor depth, lymph node metastasis, venous invasion, and lymphatic vessel invasion. We also evaluated the association with the prognostic nutritional index (PNI), modified Glasgow prognostic score (mGPS), and geriatric nutritional risk index (GNRI) as prognostic markers for other additional nutritional assessments.

### 2.4. Statical Analysis

Statistical analyses were performed using JMP Pro, version 17 (SAS Institute Inc., Cary, NC, USA). Survival curves were calculated using the Kaplan–Meier method; two groups were compared using the log-rank test, univariate comparisons of patient backgrounds were made using the Pearson χ^2^ test, and univariate and multivariate analyses were made using the Cox hazard model. The significance level was set at 5%.

## 3. Results

**Figure 1:**
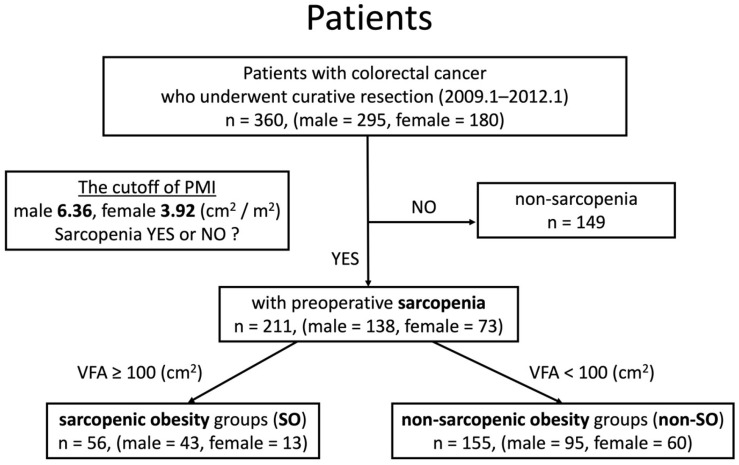
Study flow diagram. Initially, 363 patients were identified, but 3 were excluded due to incomplete clinical information. In the remaining 360 patients with colorectal cancer who underwent radical resection at our institution between January 2009 and January 2012, sarcopenia was assessed using a psoas muscle mass index (PMI) cutoff of 6.36 cm^2^/m^2^ for men and 3.92 cm^2^/m^2^ for women. After excluding 149 patients without sarcopenia, 211 patients with sarcopenia were included in this study. These patients were further divided into two groups, sarcopenic obesity (SO) (n = 56, 26.5%) and non-sarcopenic obesity (non-SO) (n = 155, 73.5%), using a visceral fat area (VFA) cutoff of 100 cm^2^ for both men and women.

**Figure 2:**
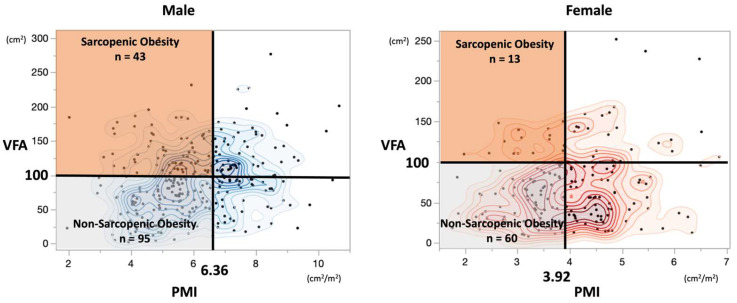
Scatterplots of visceral fat area (VFA) and psoas muscle mass index (PMI) based on sex for 360 patients (male n = 295, female n = 180) and their respective cutoffs. The VFA and PMI of 360 patients (295 males and 180 females) are presented in a scatterplot with sex-specific cutoff values. Among these, 211 patients with sarcopenia (138 males and 73 females) were categorized into two groups: 56 patients with sarcopenic obesity (43 males and 13 females) and 155 patients without obesity (95 males and 60 females).

Among the 360 patients with CRC who underwent radical resection at our institution between January 2009 and January 2012 and met our inclusion criteria, 211 patients with sarcopenia were included in the current study after excluding 149 non-sarcopenic patients. The 211 sarcopenic patients were divided into a sarcopenic obesity group (SO) (n = 56, 26.5%) and a non-sarcopenic obesity group (non-SO) (n = 155, 73.5%) (Figure 1 and Figure 2). When comparing the prognoses between the excluded non-sarcopenic group (n = 149) and the included sarcopenic group (n = 211), no significant differences were found in OS, CSS, or CRRFS (Supplementary Figure S1).

### 3.1. The Relationship of Patient Characteristics with Sarcopenic Obesity

The patient backgrounds were compared between the SO (n = 56) and non-SO (n = 155) groups. Significant differences were found between the two groups in terms of age (≥65 years, < 65 years) (*p* = 0.003), sex (*p* = 0.037), BMI (≥25, <25) (*p* = 0.031), and GNRI (≥98, <98) (*p* = 0.006). However, serum albumin (≥3.5, <3.5), CRP (≥1, <1), carcinoembryonic antigen (CEA) (≥5, <5), CA19-9 (≥38, <38), neoadjuvant chemotherapy (yes, no), pStage (0 or 1, 2 or 3), PNI (≥45, <45), and mGPS (2, 1, or 0) were not significantly different between the two groups (Table 1).

### 3.2. Comparison of Patient Prognosis between SO and Non-SO Groups

**Figure 3:**
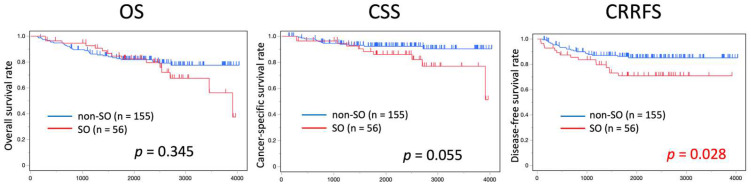
Survival curve of overall survival, cancer-specific survival, and cancer-related relapse-free survival comparing the sarcopenic obesity and non-sarcopenic obesity (non-SO) groups. Survival curve of overall survival (OS), cancer-specific survival (CSS) and cancer-related relapse-free survival (CRRFS) comparing sarcopenic obesity (SO) group and non-sarcopenic obesity (non-SO) group. Red and blue lines show SO and non-SO, respectively. CRRFS was significantly worse in patients with SO than in those without SO, and CSS tended to be worse in patients with SO than in those without SO. OS was not significantly different between the two groups.

Prognosis was compared between patients with SO (n = 56) and those without SO (n = 155) in terms of OS, CSS, and CRRFS (Figure 3). CRRFS was significantly worse in patients with SO than in those without SO. CSS tended to be worse in patients with SO than in those without SO. OS was not significantly different between the two groups.

Next, univariate and multivariate analyses with patient background factors were performed for CRRFS, which showed a significantly poorer prognosis for SO (Table 2). Univariate analysis of CRRFS showed that the following eight factors were associated with poor prognosis: CEA (≥5, <5) (*p* = 0.001), CA19-9 (≥38, <38) (*p* = 0.006), degree of differentiation (others, tub) (*p* = 0.005), depth of tumor invasion (T1 or 2, T3 or 4) (*p* = 0.004), lymph node metastasis (present, absent) (*p* < 0.001), venous invasion (present, absent) (*p* = 0.005), lymphatic vessel invasion (present, absent) (*p* = 0.007), and sarcopenic obesity (*p* = 0.037). The PNI, mGPS, and GNRI were not identified as significant prognostic factors for CRRFS. Multivariate analysis of these eight factors revealed that the following three factors were independent poor prognostic factors: CEA (≥5, <5) (*p* = 0.027), degree of differentiation (others, tub) (*p* = 0.041), and sarcopenia obesity (*p* = 0.023).

## 4. Discussion

Numerous studies have reported the impact of sarcopenia on various types of cancer, including colorectal cancer [2], pancreatic cancer [13], bladder cancer [14], gastric cancer [15], and breast cancer [16]. These studies consistently demonstrate that patients with sarcopenia have a poorer prognosis compared to those without sarcopenia. Specifically, for colorectal cancer, three meta-analyses have consistently shown that preoperative sarcopenia is linked to a higher risk of postoperative complications and reduced survival [4,5,6]. Similarly, obesity has been linked to several types of cancer, with high BMI increasing the risk of colorectal, liver, thyroid, gallbladder, ovarian, and postmenopausal breast cancers [17]. Furthermore, some studies have reported the association between obesity and prognosis in cancers such as breast cancer [18], pancreatic cancer [19], and ovarian cancer [20]. Sarcopenia and obesity are inter-related, as an increase in fat tissue leads to higher levels of inflammatory cytokines, causing muscle inflammation. Additionally, increased secretion of adiponectin and leptin reduces insulin sensitivity in the muscles, resulting in muscle mass loss [21,22]. Given these relationships, it is crucial to study sarcopenic obesity in the context of cancer using clinical data. While sarcopenic obesity has been associated with poor prognosis in pancreatic [23], liver [24], and esophageal cancers [25], the evidence remains mixed for colorectal cancer. Some studies report that sarcopenic obesity worsens recurrence-free survival (RFS) in Stage II and III colorectal cancer [26], while others have found no significant difference in disease-free survival (DFS) or overall survival (OS) after hepatic resection for colorectal liver metastasis [27]. Given this conflicting evidence, this study adds to the literature by examining the prognosis of colorectal cancer patients with sarcopenic obesity following surgery.

This study provides valuable insights into the effect of SO on the prognosis of patients with CRC. These findings suggest that sarcopenic obesity is a significant factor influencing CRRFS and CSS in patients with CRC who undergo radical resection. Although this study did not find a statistically significant difference in OS between the SO and non-SO groups, the observed trends in CRRFS and CSS highlight the importance of considering SO as a distinct clinical entity with unique prognostic implications.

The results demonstrated that CRRFS was significantly worse in the SO group than in the non-SO group. This finding was consistent with previous studies that reported a negative impact of sarcopenia on colorectal cancer prognosis [28]. Sarcopenia, characterized by loss of muscle mass and function, is often associated with increased frailty, reduced physical activity, and a higher incidence of postoperative complications. When combined with obesity, which is independently associated with an increased risk of cancer and poor outcomes, the synergistic effects of these conditions appear to exacerbate their negative impact on patient prognosis.

This study also found that CSS tended to be worse in the SO group, although this trend was not statistically significant. This suggests that while SO may contribute to a higher likelihood of cancer recurrence or progression, other factors such as the patient’s overall health status and aggressiveness of the tumor may also play crucial roles in determining long-term survival outcomes. The lack of significant differences in OS between the SO and non-SO groups may be attributed to the relatively short follow-up period or the influence of other confounding factors such as differences in treatment regimens or comorbidities.

Comparison of patient characteristics between the SO and non-SO groups revealed significant differences in the age, sex, BMI, and GNRI. Older age and a higher BMI were more prevalent in the SO group, aligning with the understanding that sarcopenia and obesity often coexist in older adults. The higher GNRI scores in the non-SO group suggest that better nutritional status is associated with the absence of obesity in patients with sarcopenia, further emphasizing the complex interplay between nutrition, body composition, and cancer outcomes.

Interestingly, serum albumin, CRP, preoperative tumor markers (CEA and CA19-9), PNI, and mGPS were not significantly different between the SO and non-SO groups. These findings indicate that traditional markers of nutritional and inflammatory status may not fully capture the prognostic implications of SO in patients with CRC. This suggests that the assessment of sarcopenia and obesity as distinct and combined conditions may provide additional prognostic information beyond conventional clinical parameters.

The identification of sarcopenic obesity as an independent poor prognostic factor for CRRFS in patients with CRC has important clinical implications. First, it underscores the need for routinely screening for sarcopenia and obesity in patients with CRC, particularly in those undergoing radical resection. The use of imaging techniques to assess muscle mass and visceral fat area, as employed in this study, should be considered in clinical practice to identify high-risk patients who may benefit from targeted interventions.

Second, the findings highlight the potential benefits of integrating nutritional and physical activity interventions in the care of patients with CRC and SO. Given the association between sarcopenia, obesity, and poor prognosis, strategies aimed at preserving or enhancing muscle mass, reducing excess body fat, and improving overall fitness could potentially improve the clinical outcomes in this patient population. Such interventions include resistance training, dietary modifications, and pharmacological approaches to modulate muscle and fat metabolism.

Third, this study raises important questions regarding the mechanisms underlying the negative impact of SO on CRC prognosis. Further research is thus needed to elucidate the biological pathways linking sarcopenia, obesity, and cancer outcomes, including their roles in systemic inflammation, insulin resistance, and altered energy metabolism. Understanding these mechanisms could facilitate the development of novel therapeutic strategies targeting specific vulnerabilities in patients with SO.

Despite the valuable insights gained from this study, several limitations must be acknowledged. The retrospective design of this study may have introduced selection bias (such as in patients with colorectal cancer who underwent radical resection), and the relatively small sample size limits the generalizability of the findings. Additionally, this study did not account for potential confounding factors, such as differences in adjuvant chemotherapy regimens, lifestyle factors, comorbidities related to obesity or sarcopenia, or genetic predispositions, which could influence patient outcomes. Furthermore, the dataset did not include data on the number of lymph nodes harvested, which may have a significant correlation with visceral fat and influence prognosis in colorectal cancer patients [29]. This is an important factor that future studies should aim to address. Muscle mass and obesity were evaluated using the PMI, but the cutoff value separating the sarcopenia and non-sarcopenia groups remains to be standardized. In this study, we used the sarcopenia evaluation standard of the Hepatological Society (6.36 cm^2^/m^2^ for males and 3.92 cm^2^/m^2^ for females), which is defined as the mean PMI minus twice the standard deviation of healthy people under 50 years of age. As the population in this study mainly comprised older patients with cancer, the use of this PMI cutoff value may have led to an overdiagnosis of sarcopenia. Another limitation is the lack of data on patients’ dietary habits, particularly protein intake, as well as changes in muscle mass during the intervention period. These factors are important for understanding the progression of sarcopenic obesity, as they can significantly influence patient outcomes. Additionally, muscle strength was not assessed in this study, although it is closely related to muscle mass and plays a crucial role in evaluating the impact of sarcopenic obesity on clinical outcomes. Furthermore, our study did not examine the relationship between muscle mass loss and circulating plasma proteins, liver protein synthesis, nitrogen balance, or protein intake in cancer patients. These factors are key to understanding changes in muscle mass, since reduced liver protein synthesis, nitrogen imbalance, or inadequate protein intake may all contribute to muscle loss in cancer patients. Future research should address these aspects to better clarify their role in sarcopenic obesity and its impact on cancer prognosis.

## 5. Conclusions

In this study, patients with sarcopenia and obesity had a shorter CRRFS than that of patients with sarcopenia but no obesity, and the presence of both sarcopenia and obesity was an independent prognostic factor for CRRFS. Thus, routine assessment of sarcopenia and obesity, coupled with targeted interventions, may improve outcomes in this high-risk population. However, further research is warranted to better understand the mechanisms underlying the negative impact of SO on cancer prognosis and to develop effective strategies for mitigating these risks.

## Figures and Tables

**Table 1:** The relationship of sarcopenic obesity with patient characteristics.

Variable (Preoperative)	SO (PMI < Median and VFA ≧ 100 cm^2^) (n = 56)	Non-SO (PMI < Median and VFA ≦ 100 cm^2^) (n = 155)	*p*-Value
Age (years, mean) (min–max)	71.3 (49–88)	66.4 (35–87)	0.003
Sex (male/female)	43/13	95/60	0.037
BMI (≥25/25>/NA)	9/47/0	10/145/0	0.031
ALB (≥3.5/3.5>/NA)	44/10/1	113/39/3	0.290
CEA (≥5/5>/NA)	5/48/3	20/130/5	0.458
CA19-9 (≥38/38>/NA)	15/41/0	42/110/3	0.903
Neoadjuvant chemotherapy (yes/no/NA)	3/53/0	14/141/0	0.387
pStage(0/1/2/3/NA) *	0/22/18/16/0	14/50/50/41	0.794
PNI (≥45/45>/NA)	29/27/0	75/79/1	0.693
mGPS (2,1/0/NA)	15/37/3	52/97/6	0.435
GNRI (≥98/98>/NA)	34/20/2	63/89/3	0.006

*: In this study, cases of colorectal cancer with pStage 4 were excluded. For the statistical analysis, the Pearson χ^2^ test was performed between the pStage 0 or 1 group and the pStage 2 or 3 group.

**Table 2:** Cox regression analysis of cancer-related relapse-free survival.

	Univariate			Multivariate		
Variable	HR	95%CI	*p*-Value	HR	95%CI	*p*-Value
Age (≥65/<65 years)	0.917	0.463–1.815	0.803			
Sex (male/female)	1.260	0.620–2.561	0.523			
Location (right/left)	0.601	0.283–1.278	0.186			
CEA (≥/5>)	3.075	1.568–6.032	0.001	2.334	1.103–4.939	0.027
CA19-9 (≥38/38>)	3.055	1.381–6.758	0.006	1.422	0.576–3.516	0.455
Degree of differentiation (others/tub)	3.589	1.487–8.659	0.005	2.839	1.043–7.729	0.041
Depth of tumor invasion (T1, 2/T3, 4)	0.324	0.152–0.690	0.004	0.640	0.238–1.716	0.375
Lymph node metastasis (present/absent)	3.068	1.596–5.900	<0.001	1.814	0.861–3.822	0.117
Venous invasion (present/absent)	2.620	1.330–5.161	0.005	1.721	0.785–3.776	0.175
Lymphatic vessel invasion (present/absent)	4.180	1.475–11.845	0.007	1.560	0.466–5.219	0.470
Sarcopenia obesity (yes/no)	2.070	1.066–4.015	0.037	2.240	1.119–4.488	0.023
PNI (≥45/45>)	0.577	0.239–1.136	0.112			
mGPS (2, 1/0)	1.867	0.948–3.676	0.071			
GNRI (≥98/98>)	1.179	0.601–2.313	0.632			

## Data Availability

The data supporting the findings of this study can be obtained from the corresponding authors upon reasonable request.

## Supplementary Materials

The following supporting information can be downloaded at https://www.mdpi.com/article/10.3390/cancers16193429/s1, Figure S1: Survival curve of overall survival, cancer-specific survival, and cancer-related relapse-free survival comparing the sarcopenia and non-sarcopenia groups.
