# Supplementary material for: Examination of Sarcopenia with Obesity as a Prognostic Factor in Patients with Colorectal Cancer Using the Psoas Muscle Mass Index
Source: Cancers (Basel). 2024 Oct 9;16(19):3429. doi: 10.3390/cancers16193429 (PMC11482590; doi:10.3390/cancers16193429)
Supplement: Supplementary file 1 [file cancers-16-03429-s001.zip › cancers-3217224-supplementary.pdf]

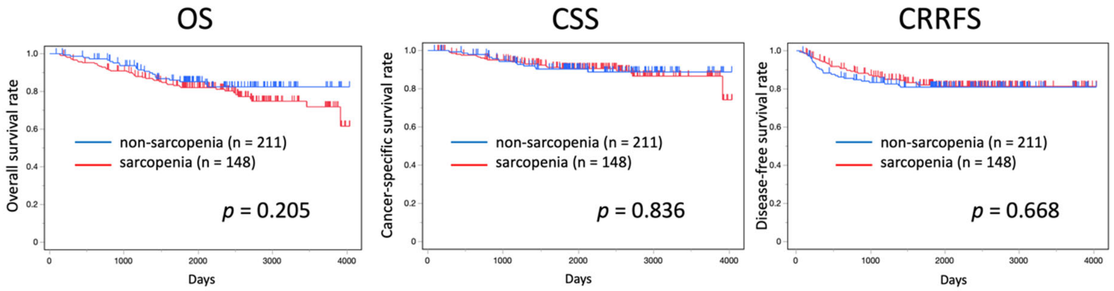

Figure S1. Survival curve of overall survival, cancer specific survival, and cancer-related relapse-free survival comparing the sarcopenia and non-sarcopenia groups.

Survival curves for overall survival (OS), cancer-specific survival (CSS), and cancer-related relapse-free survival (CRRFS) were compared between the sarcopenia and non-sarcopenia groups without considering obesity. Red and blue lines represent sarcopenia and non-sarcopenia groups, respectively. There were no significant differences in the OS, CSS, or CRRFS between the two groups.
